# Supplementary material for: Transcriptome Expression Profiling Reveals the Molecular Response to Salt Stress in Gossypium anomalum Seedlings
Source: Plants (Basel). 2024 Jan 20;13(2):312. doi: 10.3390/plants13020312 (PMC10819910; doi:10.3390/plants13020312)
Supplement: Supplementary file 1 [file plants-13-00312-s001.zip › Supplemental materials.pdf]

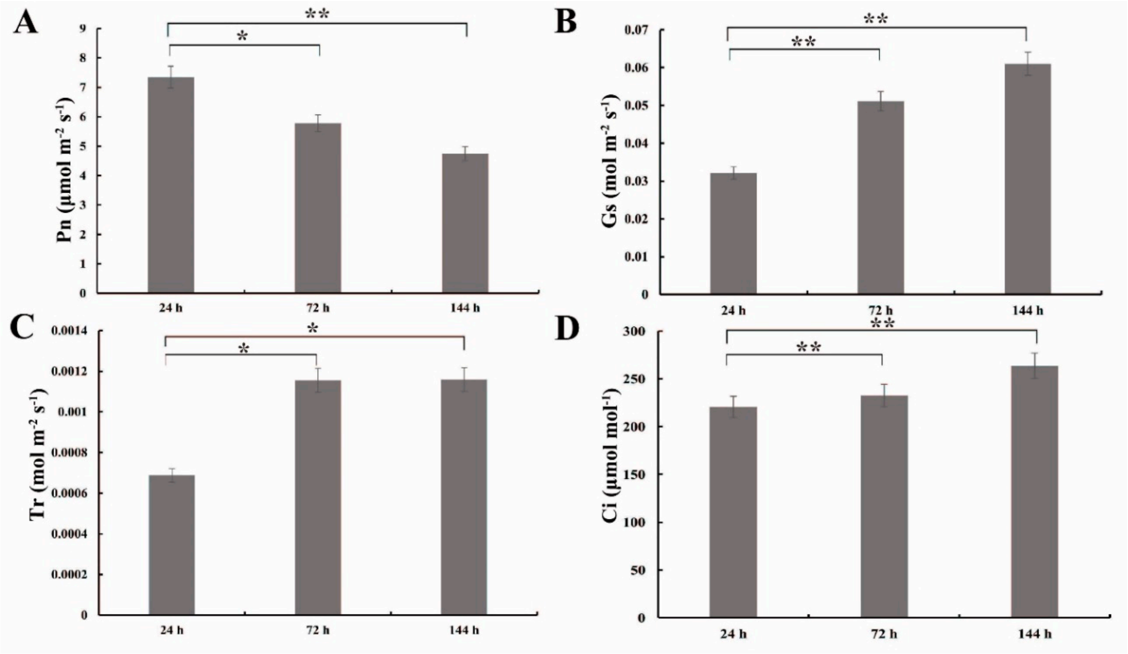

Figure S1 Photosynthetic parameters of *G.anomalum* seedlings under salt treatment. Scale bar=10cm, $*p < 0.05$, $**p < 0.01$, Student's t test. A, Pn: net photosynthetic rate. B, Gs: stomatal conductance. C, Tr: transpiration rate. D, Ci: intercellular CO_2 concentration.
